# Cost-Effectiveness of Expanding Access to Primary Health Care in Rural Rwanda by Adding Laboratory-Equipped Health Posts: A Prospective, Controlled Study

**DOI:** 10.4269/ajtmh.22-0519

**Published:** 2023-03-20

**Authors:** Donald S. Shepard, Yara A. Halasa-Rappel, Wu Zeng, Katharine R. Rowlands, Sabine F. Musange

**Affiliations:** 1 The Heller School for Social Policy and Management, Brandeis University, Waltham, Massachusetts;; 2 Population and Quantitative Health Sciences, University of Massachusetts Chan Medical School, Worcester, Massachusetts;; 3 Department of Global Health, School of Health, Georgetown University, Washington, District of Columbia;; 4 School of Public Health, University of Rwanda, Kigali, Rwanda

## Abstract

To improve access to affordable primary health care and preventive services, in 2019 Rwanda’s Ministry of Health inaugurated eight laboratory-equipped second-generation health posts (SGHPs) in the Bugesera District. Patient fees through Rwanda’s insurance system (*mutuelles*) funded most operational costs through a public–private partnership. This prospective, controlled trial evaluated the posts’ impact and cost-effectiveness. Our evaluation matched the rural cells containing these posts to eight control cells in Bugesera without formal health posts. We assessed costs using 2 years of financial data; accessed use statistics at SGHPs, health centers, and in the international literature; interviewed 1,952 randomly selected residents; conducted eight focus groups; and performed difference-in-differences regressions and survival analyses. Second-generation health posts increased primary care use by 1.83 outpatient visits per person per year (*P* < 0.0001). Of the 10 prevention indicators compared with trends, two improved significantly with SGHPs (two showed nonsignificant improvements), and one indicator experienced a significant deterioration. Second-generation health posts generated health improvements at a low cost and achieved a small, but favorable, 5% margin of revenues over financial costs. Second-generation health posts produced a very favorable incremental cost-effectiveness ratio of only $101 per disability-adjusted life year averted—only 13% of Rwanda’s per-capita gross national income. In conclusion, SGHPs improved substantially the quantity of affordable outpatient care per person. However, net impacts on quality and completeness of care and prevention, although favorable, were small. For further improvements in access and quality of care, Rwanda’s health authorities may wish to incentivize quality and strengthen coordination with other health system components.

## INTRODUCTION

Universal health coverage (UHC) aims to enable all individuals to access health-care services of sufficient quality when needed, without financial hardship. Most African countries have included UHC as a goal in their national health strategies. Achieving UHC requires overcoming many challenges.^1^^–^^3^ In 2020, 58% of the population in sub-Saharan Africa and 83% of the Rwandan population lived in rural areas.^4^ These would-be users, even if care were free at the point of service, would still face barriers as a result of limited awareness, travel expenses, and time constraints. This study evaluates Rwanda’s initiative in expanding access to health care in rural areas by creating a new type of health facility, and the implications for other parts of the country and other countries in the region.

Rwanda’s Vision 2020,^5^ the government of Rwanda’s first development strategy after the 1994 genocide, included UHC as an essential building block to the country’s overall economic development. Universal health coverage was introduced to reduce poverty and close the gap in access to health-care services. The government used several innovative strategies to improve access to health care and move toward UHC. The *mutuelle de santé* (*mutuelle*), a community-based health insurance program for persons outside the formal sector, was piloted in 1999 and was established officially in 2006, with financial and administrative autonomy in each district.^6^^,^^7^ *Mutuelle* moved to prepayment mechanisms to improve access to care and to provide financial protection. In 2021, 88% of Rwanda’s 12 million inhabitants were covered by *mutuelle*.^8^ Pay-for-performance or results-based financing, an incentive program introduced in 2002, rewards providers for achievements on the quality and value of health care. It has helped improve provider availability, facility operations, and access to HIV/AIDS services.^9^^,^^10^ The establishment of a community health program in 1995 that focused on maternal and child health care, and other evidence-based interventions, lowered Rwanda’s under-five mortality rate.^11^^–^^13^ A public–private partnership in 2016 improved access^14^ to health-care services, including access to family planning^15^ and specialized care; and strengthened supply chain management and infrastructure development.^16^^–^^18^

These initiatives helped Rwanda improve the health of its population. Rwanda’s economy also grew, with real (inflation-adjusted) income per capita (in 2019 U.S. dollars) increasing from $261 in 2000 to $820.^19^ Per-capita health-care spending increased from $9 in 2000 to $58 in 2018.^20^ In 2015, Rwanda met all health-related Millennium Development Goals. In nutrition, Rwanda achieved the target reduction in underweight children, but not in stunting because of inadequate diets.^21^ Life expectancy at birth improved from 48.6 years in 2000 to 68.7 years in 2018.^22^ In 2000, Rwanda’s maternal mortality ratio per 100,000 live births was 1,160 in Rwanda compared with 870 in sub-Saharan Africa overall. By 2018 to 2019, Rwanda had advanced from a laggard to a leader in health indicators compared with sub-Saharan Africa overall. By 2017, the maternal mortality rate in Rwanda plummeted to 248 per 100,000 births compared with 537 in sub-Saharan Africa.^23^ In 2000, the under-five mortality rate per 1,000 live births was 178.7 in Rwanda compared with 150.6 in sub-Saharan Africa. By 2019, this rate had been cut dramatically to 34.3 in Rwanda compared with 75.8 in sub-Saharan Africa.^24^

Rwanda’s health system has three major levels. The central level develops overall strategies, technical frameworks, and health policies; monitors and evaluates operational programs; and operates national referral and teaching hospitals that offer tertiary care. The intermediate level consists of referral and provincial hospitals established to relieve the burden on national referral hospitals resulting from the ever-increasing demand for specialized care. The lowest (peripheral) level consists of the 30 administrative districts. Each plans, manages, coordinates, and evaluates the district’s activities that encompass district hospitals, primary health-care (PHC) facilities (health centers and health posts), and a network of community health workers (CHWs).^25^

Despite this progress, challenges persisted in infrastructure, finance, and technical resources.^13^^,^^18^ Although PHC accounted for more than 90% of health services provided in Rwanda, 22% of Rwandans faced geographic barriers to health care, and 77% faced financial barriers in accessing health-care services, especially for specialized services. Although Rwanda’s standard was one PHC facility per 5,000 people, in 2018 the country had only one facility for 8,300 people for every PHC facility, indicating a need for scaling up the number of health facilities.^26^

In 2019, as part of a public–private partnership, Rwanda’s Ministry of Health, in collaboration with Abbott, Inc., inaugurated eight second-generation health posts (SGHPs) in rural cells in the Bugesera District (∼1 hour by road from the capital) to improve access to PHC. Second-generation health posts added a new layer of health facilities by offering the same services provided at first-generation health posts and added laboratory services, and, in some cases, maternity services and dental care. Abbott funded the buildings, equipment, initial supplies, and staff training for eight SGHPs on behalf of the Ministry of Health. For laboratory services, each SGHP was staffed with a full-time laboratory technician, and equipment and supplies to support antenatal testing and routine primary care.

Both professional and laboratory services are billed on a fee-for-service basis. Most patients are *mutuelle* members. For members of a *mutuelle* or an insurance scheme for the formal sector, the insurer pays most of the fees whereas the patient makes a small, out-of-pocket copayment. *Mutuelles* in turn are financed by a combination of household premiums, cross subsidies from formal sector schemes, and government subsidies.

This prospective controlled trial aimed to 1) assess the effect of SGHPs on access to PHC in Rwanda and the change in the quality of care, 2) examine the financial sustainability of SGHPs, and 3) evaluate the cost-effectiveness of SGHPs. The results could inform policy leaders in other sub-Saharan African countries when considering implementation of SGHPs in rural areas.

## MATERIALS AND METHODS

### Assessing the effect of SGHPs on access to quality of PCH and cost of care.

When the initiative was planned in 2018, the Bugesera District had 50 cells, the administrative unit between the sector and a village. Each cell contained several villages with a combined population of ∼6,500 persons. The district was served by the district hospital and health centers. In addition, most cells had health posts operated by a nongovernmental organization or the government. However, 16 cells had no health posts. The Ministry of Health selected eight cells without health posts and located somewhat remotely from their health center as locations for SGHPs. To evaluate this initiative, we developed a prospective, controlled cluster study design in which the cell was the unit of analysis. The eight cells selected for SGHPs constituted the intervention cells whereas the remaining eight cells without official health posts were control cells. Twelve health centers served the 16 study cells (Supplemental Table 1).

We used multiple data sources to estimate the effect of SGHPs on access to health care, including administrative data compiled by the Society for Family Health (SFH), an analysis of patient registration records from health centers serving the study cells, two rounds of household surveys, and qualitative data from focus group discussions (FGDs). In addition, we conducted interviews with key stakeholders (Dr. William Rutagengwa, Bugesera District Medical Director; and Dr. Diana Mutamba, Manager of SFH) to inform the interpretation of findings. We also used published statistical information to compare the Bugesera District with other parts of Rwanda, and conducted four FGDs (see Supplemental Appendix).

All household survey data were collected initially by interviewers circling responses on paper questionnaires. Each data collector then entered the response into a 2019 version Excel (Microsoft Corp., Redmond, WA) spreadsheet. Researchers then merged these spreadsheets into a single Excel workbook and conducted logic checks, such as checking whether each entry fell within the item’s valid range. Missing or out-of-range entries were corrected by reference to the original paper questionnaire. We next performed descriptive analyses of frequencies and means of key variables by study arm. These included receipt of antenatal care (ANC) visit, travel time, quality indexes, and services indexes.

Last, to quantify the effect of SGHPs on key outcome variables, we used a difference-in-differences (DIDs) approach to perform the analysis. The mathematical expression of the DIDs model is as follows:

$$y=\beta_{0}+\beta_{1}\text{int}+\beta_{2}\text{post}+\beta_{3}\text{int}\times \text{post,}$$

where *y* is the outcome variable, which includes initiation of the first ANC visit, the quality index for pregnant women, travel time for pregnant women, and so on; int indicates whether the individuals are in the intervention group (1 = in the intervention group with SGHPs, 0 = not in the intervention group with SGHPs); post represents whether the measurement was acquired after the SGHPs were established (1 = after SGHPs were established, 0 = before SGHPS were established); int × post represents the interaction between int and post; and *β*s are associated coefficients. In particular, *β*_3_ shows the pure effect of the SGHPs on outcome variables, demonstrating the impact from DIDs. We hypothesized that SGHPs would improve outcomes, such as such as shortening travel time, raising quality indexes, improving service indexes, and accelerating initiation of ANC. Table 1 shows the expected signs of *β*_3_ from the DIDs analysis.

**Table 1:** Study variables and the expected sign of the interaction term signifying the effect of SGHPs

Variables	Expected sign of *β*_3_
Initiation of first antenatal care	−
Perceived quality of care for pregnant women	+
Patient-reported service index for pregnant women	+
Round-trip travel time for pregnant women	−
Cost of overall round-trip cost	−
Parent-reported services index for children younger than 5 years	+
Round-trip travel time for children younger than 5 years	−
Travel cost for children younger than 5 years	−
Perceived quality index for adults 50 years and above	+
Patient-reported service index for adults 50 years and above	+
Round-trip travel time for adults 50 years and above	−
Travel cost for adults 50 years and above	−

SGHPs = second-generation health posts. The study’s service index (on a 0- to 100-poing scale) for pregnant women is the average number of recommended services reported during the first antenatal care visit, including a blood test to confirm pregnancy, and laboratory tests for malaria, HIV/AIDS, and other diseases.

All the analyses were conducted using Stata 17 (StataCorp, LLC, College Station, TX), with *P* < 0.05 used as the cutoff of statistical significance.

We used Rwanda’s Health Management Information System (HMIS) to capture the increase in routine primary care visits. The HMIS tallies the number of visits by month, site, and diagnosis at all participating health facilities, including health posts and health centers.

### Evaluating the financial sustainability of SGHPs.

The SGHPs operated as a public–private partnership. The facilities were owned by the Rwanda Ministry of Health, but were run by a private operator, usually a nurse, similar to a private clinic. The operator delivered medical services, dispensed drugs, hired and supervised up to three additional staff (e.g., a laboratory technician, additional nurses, and a medical assistant), paid salaries and other operating expenses, received revenues from patients and insurers, and provided financial and use data. The SFH provided training and compiled use and financial data for monitoring and informing the Ministry of Health.

We used these facility-based financial data to estimate the annual financial cost of providing care in an average SGHP during the first year (October 2019–September 2020) and 1 year after the establishment of the SGHPs (October 2020–September 2021). The financial cost consisted of the operations cost, including personnel and supplies, and an estimated 10% for administration. Totaling SGHPs’ monthly reports to SFH, we computed the annual revenues from services provided at the SGHPs and paid by private patients, *mutuelle* payments, and copayments. To predict the financial surplus of SGHPs, we compared the revenues to the financial cost.

In addition, we estimated the economic nonfinancial cost, which adds the annualized capital expenditures on buildings (30 years), equipment (7 years), and training (2 years) to the financial cost, and estimated the economic profit or loss as the difference between the revenues and economic cost. The capital cost was estimated for a typical SGHP and excluded the cost of a dental clinic and maternity services, which had been installed in only a few SGHPs. The buildings for each of the four SGHPs without maternity areas cost $43,000; buildings with maternity areas cost $64,000 each. Equipment (including laboratory equipment and furnishings) averaged $13,000 per SGHP. Second-generation health posts also benefited from use of the land on which the buildings were constructed, and ongoing monitoring and support from the SFH. Because of data limitations, we did not value these two inputs, but their annual value is likely small.

### Determining the cost-effectiveness of SGHPs.

Determining the cost-effectiveness of SGHPs entailed estimating the effect not only of the services they provide, but also how they impact services at other facilities in Rwanda’s health system. Second-generation health posts can potentially affect both the quantity and quality of primary care services. By seeking to bring quality services closer to the population, SGHPs can potentially increase access to primary care for routine curative services (Supplemental Figure 1).

To quantify this impact, we relied on a systematic cost-effectiveness analysis of quality services for one of Rwanda’s major health problems: malaria.^27^ That analysis compared drug treatment of malaria against no intervention. We used the authors’ central estimate as our base case, based our sensitivity analysis around its range of estimated values, and converted its results from 2012 to 2020 U.S. dollars using the U.S. gross domestic product (GDP) deflator.^28^

We extended the malaria model to another major primary care problem: acute respiratory infections (ARIs), including both upper and lower respiratory conditions. When a patient with a febrile illness seeks medical care, the clinician will not immediately know whether the problem is malaria, a respiratory illness that might respond to antibiotics, or some other condition. The preferred treatment strategy is to use a rapid test to test and treat for malaria if positive, but otherwise treat with an antibiotic as a first-line attempt to manage the illness.^29^ Because malaria and ARIs are managed by the same personnel in SGHPs, we assumed their costs and success rates per case would be comparable. Aggregate values would depend on the number of cases.

To estimate baseline levels of health burden, we used data for Rwanda for 2019 (the latest data available) from the Institute of Health Metrics and Evaluation.^30^ We did not want to limit consideration of disease burden to cases treated in and reported by the formal medical sector—the facilities covered by the HMIS. Rather, our aim was to estimate the overall disability-adjusted life years (DALYs), including patients who did not seek care or who sought care outside the formal health sector, such as CHWs. The Institute of Health Metrics and Evaluation includes cases in all settings, including self-treatment, CHWs, and no treatment. We estimated the baseline burden per case for malaria, ARIs, and intestinal parasites by dividing the estimated DALYs from each of these three conditions by the incidence of each condition. We then derived a weighting factor for ARIs and intestinal parasites as its burden per case relative to that for malaria.

Our cost-effectiveness analysis was based on results of systematic reviews published by the World Bank.^27^ These systematic reviews synthesized findings primarily from published efficacy studies, where the care was managed by researchers. However, the SGHPs in Rwanda lacked the additional funding, expertise, and monitoring available in research studies. To address this issue, we adjusted for the variation in quality—therefore effectiveness—by incorporating a percentage quality score (on a 0- to 100-point scale) for care during the follow-up period by multiplying this score (converted to a percentage) by the weighted share of visits. The product is the quality-adjusted weighted share of visits for which care was delivered correctly.

A key question was whether the episodes treated at SGHPs would otherwise have been treated by alternative providers. Such options included CHWs, who can perform some rudimentary primary care services and operate throughout the Bugesera District, and professional health center staff. We performed DID analyses to examine substitution from health center treatment. The cost-effectiveness of SGHPs, expressed as the cost per DALY averted, was calculated by dividing the annual expenditures of SGHPs per person by the DALYs saved from malaria and ARI health-care services provided at SGHPs.

## RESULTS

### Impact on access and quality of PHC, and cost of care.

#### Use of primary care services.

As expected, the household survey reported an increase in the share of visits to health posts in intervention clusters compared with control clusters for children younger than 5 years. As all distributions had to sum to 100%, the shares to health centers and CHWs were reduced (Supplemental Table 2). However, these percentage shares do not show the absolute numbers. Of the 113,788 outpatient services provided in the intervention cells, 88% were provided at the SGHPs (Supplemental Table 3). The majority of care for respiratory infections (96%), simple malaria (92%), and treatment of intestinal parasites (96%) was provided by the SGHPs. Indeed, as shown by the insignificant *P* values for interaction terms (well over 0.05), the SGHPs did not have any significant impact on the number outpatient visits, the number of ANC visits, or the number of deliveries at health centers located in the intervention cells’ catchment areas (Supplemental Table 4). This result indicates that SGHPs largely complemented and did not substitute for services provided at health centers.

Table 2 compares outpatient, ANC, and delivery services between intervention areas (combining services provided at health centers with services provided through SGHPs) to services provided in the control areas. The results confirm that SGHPs complemented health centers for outpatient services (substantially increasing total outpatient use), but had no measurable effect on ANC or deliveries. The highly significant interaction coefficient of 1,828.00 per 1,000 persons (*P* < 0.001) means that SGHPs increased outpatient use by 1.83 outpatient visits per person per year.

**Table 2:** DIDs for services in the intervention and control areas provided at both health centers and SGHPs

Variables	Estimate	SE	95% CI	*P* value
Outpatient visits per 1,000 persons				
Intervention (reference: control areas)	−65.75	321.25	−723.80 to 592.30	0.839
Time: After SGHPs (reference: before SGHPs)	−229.38	321.25	−887.43 to 428.68	0.481
** Time × intervention**	**1,828.00**	**454.32**	**897.37 to 2,758.63**	**< 0.001**
Intercept	668.00	227.16	202.69 to 1,133.31	0.007
No. of antenatal care visits				
Intervention (reference: control areas)	199.63	86.79	21.84 to 377.41	0.029
Time: After SGHPs (reference: before SGHPs)	−108.38	86.79	−286.16 to 69.41	0.222
**Time × intervention**	**66.25**	**122.74**	−**185.18 to 317.68**	**0.594**
Intercept	356.75	61.37	231.04 to 482.46	< 0.001
No. of deliveries				
Intervention (reference: control areas)	89.13	43.17	0.69 to 177.56	0.048
Time: After SGHPs (reference: before SGHPs)	−12.13	43.17	−100.56 to 76.31	0.781
**Time × intervention**	−**0.75**	**61.05**	−**125.81 to 124.31**	**0.990**
Intercept	112.13	30.53	49.60 to 174.65	< 0.001

DIDs = difference in differences; SE = standard error; SGHPs = second-generation health posts. Key interactions for assessing the effect of SGHPs are in bold type.

#### Promptness in initiating ANC.

The timing of the first antenatal visit was reported in the household surveys. Of the 2,016 individuals invited to participate in the interviews, 1,952 (96.8%) responded to the survey. Of the 1,952 respondents, 655 were pregnant women, 656 were mothers of children younger than 5 years old, and 641 were adults 50 years or older (Supplemental Figure 2).

**Figure 1.**
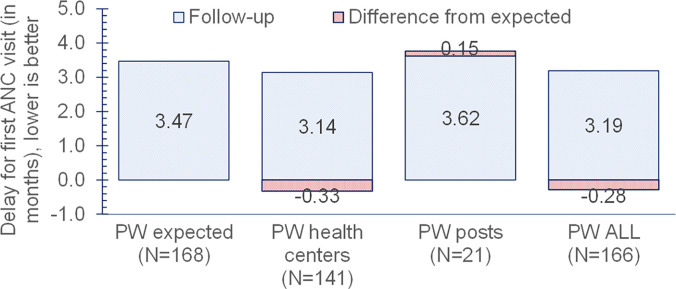
Delay in seeking first antenatal care (ANC) visit by type of site. posts = second-generation health posts; PW = pregnant women.

At baseline, women in the intervention areas initiated their first ANC visit within a mean ± SD of 3.7 ± 1.6 months of their last menstrual period compared with 3.0 ± 1.4 months in the control area. This pattern was consistent with the greater remoteness of intervention sites. After implementing SGHPs, pregnant women in the intervention areas sought their first ANC visit within 3.2 ± 1.4 months of their last menstrual period compared with 2.8 ± 1.3 in the control areas. The expected delay in initiating the first ANC visit for women in the intervention areas was 3.47 months based on trends in control areas (Figure 1). Compared with the expected trend without the SGHPs, the time to the first ANC visit decreased by 0.28 month or ∼1.23 weeks in the intervention areas (*P* = 0.21). When separated by the site of the first ANC visit, the delay decreased by 0.33 month or ∼10 days for women who sought ANC at health centers, and increased by 0.15 month or ∼4.5 days for women who sought care at SGHPs.

#### Quality of care and service completeness.

The indices for quality of care and service completeness showed mixed findings. Quality of care with regard to pregnant women in intervention clusters improved significantly compared with expectations from the control area. From the average follow-up score of 30.02 points (on the 0- to 100-point scale), 7.61 points (25%) was a net improvement related to the intervention. However, for adults, quality showed a nonsignificant deterioration. The service index of health-care intensity showed an improvement for pregnant women, but deterioration for children and older adults (Supplemental Appendix).

#### Travel time.

At baseline, pregnant women’s recollection of their average round-trip time seeking their first ANC visit in the intervention areas was 3.3 ± 1.1 hours compared with 3.2 ± 1.2 hours in the control area. After implementing SGHPs, pregnant women’s average round-trip time seeking their first ANC visit in the intervention areas decreased to 3.0 ± 1.1 hours. It remained the same in the control areas (3.2 ± 1.3 hours). The expected round-trip time in the intervention areas was 3.39 hours (Supplemental Figure 3). However, the actual round-trip time was 0.41 hour (25 minutes) less compared with what would have happened without SGHPs. The reduction was greater for pregnant women seeking their first ANC visit in the SGHPs (0.7 hour, or 42 minutes) compared with the health centers (0.4 hour, or 23 minutes).

At baseline, the average round-trip time for children younger than 5 years seeking health care in the intervention areas was 1.1 ± 0.9 hours compared with 1.5 ± 1.3 hours in the control area. After implementing SGHPs, the average round-trip time for children younger than 5 years seeking health care in the intervention areas decreased to 1.0 ± 0.7 hour and 1.2 ± 1.0 hours in both the intervention and control areas, respectively. Although the expected round-trip time in the intervention areas for children was 0.84 hour (Supplemental Figure 3), the actual round-trip time was 0.11 hour (7 minutes) longer compared with what would have happened without SGHPs. Further analyses found this was a result of a selection effect; the increase in travel time occurred among those seeking care in health centers (0.19 hour or 11 minutes).

For adults age ≥ 50 years, the average round-trip time for seeking care in the intervention areas at baseline was 1.9 ± 1.1 hours compared with 1.8 ± 1.2 hours in the control areas. After implementing SGHPs, the average round-trip time for adults age ≥ 50 years seeking care in the intervention areas decreased to 1.7 ± 0.9 hours and increased to 2.0 ± 1.1 hours in the control areas. The expected round-trip time in the intervention areas for adults was 2.1 hours (Supplemental Figure 3). However, the actual round-trip time was 0.41 hour (25 minutes) less compared with what would have happened without SGHPs. The reduction was greater among those seeking care at the SGHPs (0.56 hour, or 34 minutes) compared with the health centers (0.24 hour, or 14 minutes).

#### Time to seeking care from illness onset.

**Figure 2.**
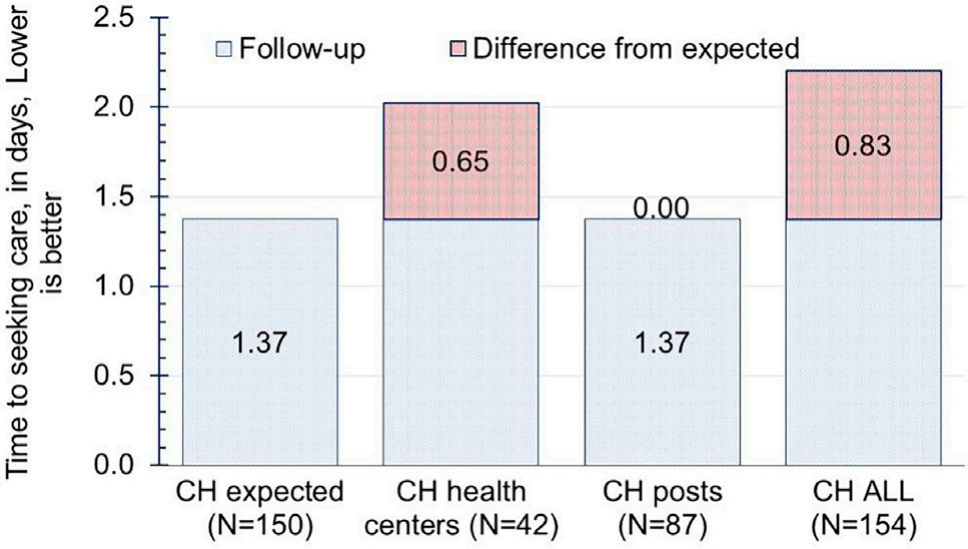
Time to seeking care for children younger than 5 years from illness onset by site (days). CH = child; posts = second-generation health posts.

At baseline, promptness in seeking health care for children younger than 5 years from illness onset was 2.3 ± 1.4 days in the intervention areas compared with 2.8 ± 4.7 days in the control areas. After implementing SGHPs, the promptness of seeking care improved to 2.2 ± 3.2 days and 1.9 ± 0.7 days in both the intervention and control sites, respectively. The expected improvement in time to seeking care from illness onset in the intervention areas was 1.37 days (Figure 2). However, the overall time to seeking care increased by 0.83 day. This increase was observed for care sought at health centers (0.65 day), whereas no changes occurred in seeking care at SGHPs. Table 3 shows that the establishment of SGHPs was associated with improvements in 5 of the 10 indicators.

**Table 3:** Summary of survey-based study indicators

Indicator	Improved*	Impact	*P* value
Pregnant women			
Promptness in receiving antenatal care, weeks	Yes	1.23	0.212
Perceived quality index, 1–100 points	Yes†	7.61	0.001
Service index, 1–100 points	Yes	2.26	0.358
Improvement in round-trip travel time, hours	Yes†	−0.41	0.028
Children younger than 5 years			
Promptness in seeking care from illness onset, days	No	0.83	0.085
Service index, 1–100 points	No†	−4.61	0.012
Improvement in round-trip travel time, hours	No	0.11	0.477
Adults ≥ 50 years			
Perceived quality index, 1–100 points	No	−1.97	0.446
Service index, 1–100 points	No	−6.34	0.134
Improvement in round-trip travel time, hours	Yes†	−0.41	0.018

* Yes indicates a favorable change; No indicates an unfavorable change.

† Change is statistically significant (*P* < 0.05).

#### Interpretation of FGDs.

Focus group discussions identified a number of organizational challenges regarding relationships among district health staff, CHWs, health post operators, and health centers (Supplemental Appendix). Discussions among the stakeholders have been addressing these challenges.

### Financial sustainability of SGHPs.

On average, the financial cost of running a typical SGHP during the establishment year, October 2019 to September 2020, was $12,217 (Table 4). The financial analysis focused on these months to include the initial inventory investments in October 2019 and to avoid a bias in favor of the SGHPs. The average revenues at a typical SGHP during the establishment year was $15,400, generating a financial surplus of $3,183 (21% of revenues). The economic analysis incorporated in-kind subsidies to the SGHPs from donors and the government for the building, equipment, and initial training. The economic cost of a typical SGHP during the same period was $17,469, of which $5,252 was for annualized capital cost. From the economic perspective, a typical SGHP operated at an economic loss of $2,069 (13% of revenues) during that year. In the second year of establishment, October 2020 through September 2021, revenues rose by 45% whereas costs grew by only 8%. As a result, the financial surplus grew to 41% of revenues, and the economic balance turned from a 13% deficit to a 17% profit.

**Table 4:** Financial and economic analysis of a typical SGHP

Component and formula	October 2019–September 2020		October 2020–September 2021	
	Annual amount, $	Percentage of revenues	Annual amount, $	Percentage of revenues
Revenue	15,400	100	22,319	100
Financial costs	12,217	79	13,244	59
Financial surplus: Revenue – Financial costs	3,183	21	9,075	41
Annualized capital cost*	5,252	34	5,252	24
Economic costs: Financial costs + Annualized capital cost	17,469	113	18,496	83
Economic profit or loss: Revenues − Economic costs	−2,069	−13	3,823	17

SGHP = second-generation health post.

* Capital cost is based on the limited second-generation health post model, which excludes the dental clinic and maternity ward.

### Cost-effectiveness of SGHPs.

Table 5^31^^,^^32^ presents the estimated cost-effectiveness of SGHPs. The malaria incremental cost-effectiveness ratio, adjusted to 2020 prices, was $6.77 per DALY averted. Factoring in ARIs, intestinal parasites, other outpatient services, and the quality of services derived from our household survey, we estimated the cost-effectiveness of general outpatient services at $65 per DALY averted. Our sensitivity analysis found a 95% CI of $17 to $157. These amounts would generate 1 additional year of good health as a result of SGHPs. All of these values are substantially less than Rwanda’s GDP per capita of $798 in 2020.^33^ Using the guideline of the WHO Commission on Macroeconomics in Health, this makes these outpatient services highly cost-effective.^34^

**Table 5:** Derivation of cost-effectiveness of routine outpatient services provided by SGHPs

Description	Malaria	ARI	Intestinal parasites	All outpatient	Source or formula
Malaria case management vs. no intervention					
Malaria ICER estimate for 2012, cost/DALY averted	$5.96	–	–	–	Tediosi et al.^27^
Malaria ICER range for 2012, cost/DALY averted	$1.55–$14.38	–	–	–	Tediosi et al.^27^
Relative range of malaria ICER*	0.260–2.413	–	–	–	Malaria ICER range for 2012/Malaria ICER estimate for 2012
U.S. inflation adjustment for 2012 to 2020	1.1358	–	–	–	World Bank^28^
Malaria ICER estimate for 2020, cost/DALY averted	$6.77	–	–	–	U.S. inflation adjustment for 2012 to 2020 × Malaria ICER estimate for 2012
Adjustment to all outpatient services					
No. of Bugesera-adjusted SGHP visits November 2019 to October 2020	6,867	26,469	18,924	100,303	–
Bugesera visit share in SGHPs, %	6.85	26.39	18.87	100.00	Musange^31^
Rwanda national incidence for 2019, no. of cases	695,856	5,563,236	3,055,329	–	Institute for Health Metrics and Evaluation,^30^ Izere and Neel^32^
Rwanda national DALYs for 2019	251,111	290,116	15,834	–	Institute for Health Metrics and Evaluation^30^
Disability weight by condition, DALYs/incidence	0.3609	0.0521	0.0052	–	Rwanda national DALYs for 2019/Rwanda national incidence for 2019
DALY weight for merging ARI†	1.0000	0.1445	0.0144	–	Disability weight by condition for ARI /Disability weight by condition for malaria
Malaria DALY-weighted visit share in the Bugesera District, %	6.85	3.81	0.27	10.93	Bugesera visit share in SGHPs × DALY weight for merging ARI
Average service quality in SGHPs	0.952	0.952	0.952	0.952	Supplemental Figure 5
Quality-adjusted weighted visit share in the Bugesera District, %	6.52	3.63	0.26	10.41	Malaria DALY-weighted visit share in the Bugesera District × Average service quality in SGHPs
Malaria-equivalent episodes per outpatient visit, %	–	–	–	10.41	Overall quality-adjusted weighted visit share in the Bugesera District
Outpatient ICER estimate for 2020, $/DALY averted	–	–	–	$65	Malaria ICER estimate for 2020/Malaria-equivalent episodes per outpatient visit
Range of outpatient ICER for 2020, $/DALY averted	–	–	–	$17–$157	Outpatient ICER estimate for 2020 × Relative range of malaria ICER

ARI = acute respiratory infection (both upper and lower); DALY = disability-adjusted life year; ICER = incremental cost-effectiveness ratio; SGHP = second-generation health post.

* The best estimate = 1.00.

† Malaria = 1.00.

## DISCUSSION

### Impact on access to care.

The administrative data suggest that SGHPs complemented health centers’ provision of outpatient care for conditions such as ARIs, injuries, and oral health care. However, we did not find a significant change in the provision of ANC services. For outpatient visits, for all intervention areas combined, the SGHPs showed a remarkably high penetration rate during their first full year of operation (November 2019–October 2020). The use data showed that 88.1% of the 113,788 outpatient visits in all the intervention areas took place in SGHPs. From the household survey, we estimated some modest improvements, but they were not statistically significant. The delay in seeking the first ANC visit was reduced in the intervention areas by about a week. Women sought care 1.4 weeks earlier at health centers, but 0.6 week later in SGHPs in the intervention areas compared with trend-based expectations.

The quality of health care in the intervention areas improved by 7.6 points for pregnant women (on a 0- to 100-point scale). Findings from the FGDs suggest that the small staff size of a SGHP compared with a health center, staff shortages, and staff turnover may have contributed to perceptions of declining quality of care from 2019 to 2021 at both health centers and SGHPs. On the other hand, quality of care for pregnant women improved in intervention areas compared with control areas, and the service index in the intervention areas improved by 2.3 points compared with the pre-SGHPs period. Travel time to a health post (averaging 17 minutes one way) was shorter than to a health center per administrative data compiled by the SFH.

In household surveys, respondents reported much longer travel times than the administrative data suggested. The FGDs offered several explanations for this discrepancy. First, because the women were pregnant, some were not able to walk. They had to walk slowly or wait by the side of the road until an available bicycle taxi came by that could transport them. Second, respondents misinterpreted the question and incorporated time at the facility as part of their response. Third, respondents did not generally wear wristwatches or carry phones (which often remained with the household head), so they had trouble estimating time precisely, or performing the arithmetic to convert one-way time in minutes to a round trip in hours. Although the times differed among the three populations, likely because of one or more of the reasons just mentioned, patterns within a population were consistent and plausible, raising confidence that the SGHPs lowered travel time.

Poor sanitation and limited access to water are known risk factors for intestinal parasitic infections across all ages. In Rwanda, rural areas experienced a greater burden than urban areas.^32^^,^^35^ Our study demonstrated that access to SGHPs addressed a high demand for curative outpatient services in a rural district of Rwanda. Rwanda is clearly a low-income country.^22^ Statistical data comparing rural and small-town Rwanda with major cities showed that the Bugesera District was poorer than the country’s major urban areas. Considering all these findings, SGHPs have contributed to greater equity in health-care access within Rwanda and offer the potential to do so globally.

As small private enterprises, the SGHPs were incentivized financially to generate a high volume of visits, with associated revenues to cover their costs and hopefully a margin for the operator. On this measure, they succeeded, averaging 1.83 visits per person per year. With no financial incentive for quality of care, preventive services, or coordination with other parts of the health system, the lack of impact was not surprising.

Despite several successes, SGHPs have not yet met their targets for various preventive indicators, such as earlier ANC visits and greater use of preventive services. There were several reasons. Some women preferred receiving ANC at a health center so the location would be familiar when they gave birth. Some policies designed to facilitate continuity of care for pregnant and postpartum women acted as unintended barriers against the use of SGHPs. For example, health centers, but not SGHPs, could provide pregnant women with insecticide-treated bed nets and, if eligible, food supplements. In addition, Rwanda’s pay-for-performance mechanism incentivized CHWs to bring pregnant women to a health center, but not a SGHP, to start ANC within the first trimester. This design created an unintended conflict between the SGHPs and the health centers, leading CHWs to stop referring most pregnant women to SGHPs. The only exceptions were women more than 3 months into their pregnancy and late in initiating ANC. In this case, the CHWs referred the woman to the SGHPs for ANC so she could avoid paying a fine (reprimand) for the delay in seeking care.

### Financial sustainability.

The SGHPs were established and evaluated during a global pandemic; however, the number of services provided during the establishment year, especially outpatient visits, exceeded those provided at the health centers. An average SGHP was financially profitable even during its first (establishment) year. By the second year, it was also profitable on economic grounds, including the value of the in-kind subsidies. Rwanda’s system of UHC ensures that SGHPs should continue to have an adequate revenue stream indefinitely as long as they continue to deliver quality care efficiently. As of mid 2022, almost 3 years since the SGHPs were established, they remain in operation and, to our knowledge, are financially viable.

The FGDs and survey data identified several areas where the SGHPs could fill gaps in health services: 1) access to iron tablets and insecticide-treated nets for pregnant women; 2) misoprostol for women delivering at home; 3) case management services and medication for chronic conditions, especially hypertension (currently covered only at health centers and higher levels), for adults ≥ 50 years; 4) laboratory tests for diagnosing intestinal parasites; 5) facility-based deliveries in lieu of at-home deliveries; 6) screening of women for cervical cancer, children for hepatitis B infection, and adults ≥ 50 years for hypertension; 7) increasing patients’ privacy, especially for women delivering at SGHPs; and 8) ensuring the availability of needed materials and equipment.

Preventive services often require outreach services and face difficulties in attracting user payments. It would be quite sensible, however, for the government of Rwanda and donors to subsidize SGHPs, particularly in their provision of preventive services, as contributions to Rwanda’s public health goals. In the same way, other public institutions, such as CHWs, health centers, and schools receive public subsidies for their contributions to important societal goals.

### Cost-effectiveness.

Our evaluation of the cost-effectiveness of SGHPs focused only on the three most common conditions: ARI, malaria, and intestinal parasites. The cost per DALYs averted as a result of these conditions provided at the SGHPs was $101, indicating that SGHPs are highly cost-effective compared with Rwanda’s GDP per capita of $798 in 2020.

Because SGHPs proved cost-effective in the Bugesera District, policymakers may wish to examine their potential expansion both within Rwanda and to other sub-Saharan African countries. Within Rwanda, any part of a district that is not well served by the existing health-care infrastructure would be a good location. These are locations relatively far from existing health centers requiring considerable time and/or expense to access care, but with a sufficient population size to make an SGHP financially viable for the operator.

When considering expansion to other countries, the WHO Health Systems Building Blocks provides a useful framework.^36^ It notes that health financing is one of the key building blocks. Rwanda satisfied this building block through its system of *mutuelles*. Second-generation health posts were able to invoice the client’s *mutuelle* for each routine primary care service. Second-generation health posts operating under Rwanda’s system of *mutuelles* achieved two important goals. For clients, they provided quality care, were close geographically, and were convenient (with laboratories, advice from a medical professional, and pharmaceutical products all available at a single location). For the SGHP operators, the *mutuelle* payments and clients’ copayments provided a reliable source of financing. After the volume increased in the Bugesera District in the year after establishment, its SGHPs became financially viable, so they should be viable in other similar districts. In May 2022, the Rwanda Ministry of Health inaugurated additional SGHPs in remote areas in Southern Province near the town of Huey.

Because Rwanda’s system of *mutuelles* was a key enabling factor for the financial viability of SGHPs, the most promising countries are ones with reasonable levels of population-based insurance coverage, especially in rural areas. Although a number of countries have coverage for formal-sector workers and their families, those persons already tend to have adequate access to health care by living in major towns or by being served by the health system of a major enterprise, such as a mine. Across 36 sub-Saharan African countries, insurance coverage averaged only 7.9%. Only four countries had estimated coverage levels with any type of health insurance above 20% (Rwanda, 78.7% [95% CI, 77.5–79.9]; Ghana, 58.2% [95% CI, 56.2–60.1]; Gabon, 40.8% [95% CI, 38.2–43.5]; and Burundi, 22.0% [95% CI, 20.7–23.2]).^37^

Other countries interested in SGHPs may need a different financing model. This could be based on some system of results-based financing with incentive payments from governments and/or donors, or direct grants from the national government. Although our favorable cost-effectiveness results indicate that government funding can be economically sound in principle, a budget impact analysis is needed to assess the feasibility of government funding in practice. If existing health facilities do not appear to be funded adequately, additional facilities could be expected to suffer similar shortfalls and would not fulfill their public health objectives. However, if a country’s publicly funded facilities have adequate supplies, functioning equipment, and reliably paid staff, then it is likely that the country could afford the modest increases needed to support SGHPs.

## CONCLUSION

Second-generation health posts were introduced and implemented as an additional layer of the health-care system in Rwanda to improve access to health-care services in rural areas. Our results support the importance of this new type of provider in rural areas, where 88% of the outpatient visits occurred at SGHPs. Our analysis indicates that most of the care provided during these visits would not have occurred without the SGHPs. Second-generation health posts’ main benefit was a substantial increase in the use of curative primary care for conditions such as malaria and ARIs. Compared with our estimate that conditions would not have otherwise received treatment, this impact was highly favorable. Second-generation health posts are one of several promising options to improving access to primary care (Supplemental Appendix).

So far, SGHPs have not achieved their goals for increasing use of preventive services. For pregnant women, Rwanda’s newly approved National Antenatal Care Guidelines^38^ may offer a promising solution. Based on policy from the WHO, the guidelines recommend that each pregnant woman receive eight contacts. Rwandan health authorities recommend that the first contact occur at a health center so that the woman can receive an ultrasound. We recommend that a portion of the subsequent visits be accessed at either an SGHP or a health center, as the woman prefers. To meet this need, we recommend that SGHPs continue to offer ANC. Last, we note that financial incentives to providers and clients have proved efficacious and cost-effective in increasing preventive and other key activities in Rwanda^39^ and globally.^40^^,^^41^ Building on this experience, we recommend that the Ministry of Health and district health offices strengthen coordination, and design and align incentives for CHWs, SGHPs, health centers, and clients to increase prevention and other key services.

## Financial Disclosure

This study was supported by research agreements from Alere International Limited, an Abbott Company, to Brandeis University and the School of Public Health, University of Rwanda. S. F. M. received support from a research agreement from Abbott, Inc., to the School of Public Health, University of Rwanda. The funding bodies had no role in the design of the study; in the collection, analysis, and interpretation of data; nor in writing the manuscript.

## Supplemental Materials


Supplemental materials
