# Supplementary material for: Cost-Effectiveness of Expanding Access to Primary Health Care in Rural Rwanda by Adding Laboratory-Equipped Health Posts: A Prospective, Controlled Study
Source: Am J Trop Med Hyg. 2023 Mar 20;108(5):1042–51. doi: 10.4269/ajtmh.22-0519 (PMC10160894; doi:10.4269/ajtmh.22-0519)
Supplement: Supplementary file 1 [file tpmd220519.SD1.pdf]

SUPPLEMENTARY APPENDIX

Cost-Effectiveness of Expanding Access to Primary Health Care in Rural Rwanda by Adding Laboratory-Equipped Health Posts: Controlled Trial

Donald S. Shepard[1*], Yara A. Halasa-Rappel[1], Wu Zeng[2], Katharine R. Rowlands[1], Sabine F. Musange[3]

[1]Institute for Global Health and Development, The Heller School for Social Policy and Management, Brandeis University, Waltham, MA, United States
[2]Department of Global Health, School of Health, Georgetown University, Washington DC, United States
[3] School of Public Health, University of Rwanda, Kigali, Rwanda

*Corresponding Author: Donald S. Shepard, PhD, The Heller School for Social Policy and Management, Brandeis University, Waltham, MA 02454 USA, shepard@brandeis.edu, +1-617-584-6664

Table of Contents

Supplemental tables and figures 2
Detailed data sources 5
Quality of care and service completeness 7
Interpretation from Focus Group Discussions 10
Comparative options for strengthening access to primary care 11
References 12

Supplemental tables and figures

Supplemental Table 1. List of cells included in the study by study arm and health center

No.	Health centers	Intervention cells	Control cells
1	Gashora	Ramiro	
2	Rweru	Batima	
3	Juru	Musovu	Kabukuba
4	Shyara		Kamabuye, Nziranzinza, Nziranzinza, Rutare
5	Mayange	Mbyo	Kibirizi
6	Ngeruka	Murama	
7	Kamabuye		Nyakayaga
8	Nyarugenge		Murambi
9	Ruhuha	Gikundamvura	
10	Mwogo	Kagasa	
11	Musenyi	Rulindo	
12	Rilima		Ntarama

Supplemental Table 2. Percentage distribution of the most recent treatment site for children under age 5

	Control arm			Intervention arm			Diff. in
Site	Before	After	Diff.	Before	After	Diff.	Diff.
Hospital	2.5	0.6	-1.9	2.5	2.6	0.1	2.0
Health Center	61.0	59.9	-1.1	61.0	27.3	-33.7	-32.6
Health Post	12.6	18.0	5.4	12.6	56.5	43.9	38.5
CHW	12.6	18.6	6.0	12.6	12.3	-0.2	-6.2
Pharmacy	7.6	1.2	-6.4	7.6	1.3	-6.3	0.1
Other	3.8	1.8	-2.0	3.8	0.0	-3.8	-1.8
Total	100.0	100.0	0.0	100.0	100.0	0.0	0.0

Notes: Diff denotes difference (after – before); CHW denotes community health worker; Diff in diff denotes difference in differences (intervention difference – control difference). Aggregate n = 647.

Supplemental Table 3. Outpatient visits in the intervention cells by facility type and condition

Facility type	All outpatient visits*	Acute respiratory diseases	Malaria	Intestinal parasites
Health Center	13,485	1,232	574	845
SGHPs	100,303	26,469	6,867	18,924
% allocated to SGHPs	88%	96%	92%	96%
Total	113,788	27,701	7,441	19,769

*Outpatient visits exclude maternal child health services. Notes: SGHP denotes second generation health posts. Data cover the year from November 2019 through October 2020.

Supplemental Table 4. DIDs for services in intervention and control areas provided at health centers only

	Estimate	SE	95% CI		p value
Outpatient visits per 1,000 persons					
Intervention (Reference: control areas)	-229.38	127.09	-489.71	30.96	0.0820
Time: Post SGHPs (Reference: pre SGHPs)	-65.75	127.09	-326.09	194.59	0.6090
**Time x Intervention**	**-158.00**	**179.74**	**-526.17**	**210.17**	**0.3870**
Intercept	668.00	89.87	483.91	852.09	0.0000
Number of ANC visits					
Intervention (Reference: control areas)	199.63	84.81	25.90	373.35	0.0260
Time: Post SGHPs (Reference: pre SGHPs)	-108.38	84.81	-282.10	65.35	0.2120
**Time x Intervention**	**-55.25**	**119.94**	**-300.93**	**190.43**	**0.6490**
Intercept	356.75	59.97	233.91	479.59	0.0000
Number of deliveries					
Intervention (Reference: control areas)	89.13	41.05	5.04	173.21	0.0390
Time: Post SGHPs (Reference: pre SGHPs)	-12.13	41.05	-96.21	71.96	0.7700
**Time x Intervention**	**-59.00**	**58.05**	**-177.92**	**59.92**	**0.3180**
Intercept	112.13	29.03	52.67	171.58	0.0010

Notes: ANC denotes antenatal care; CI denotes confidence interval; DID denotes difference in differences; SE denotes standard error; SGHP denotes second generation health posts key interaction terms for assessing impact of SGHPs **bolded**.

Supplemental Figure 1. Percent of visits for the first 10 diagnoses at health centers in patients from the control and intervention areas before and during the introduction of SGHPs.

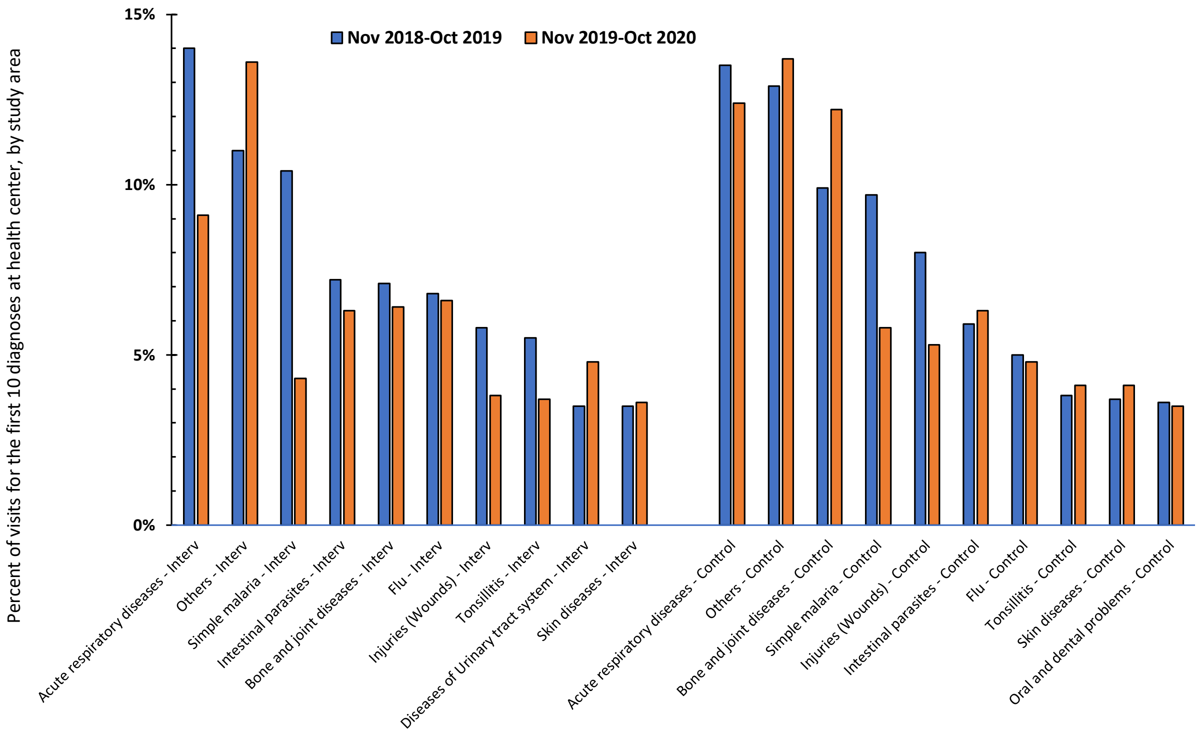

Supplemental Figure 2. Response rate by population type, N=1,952

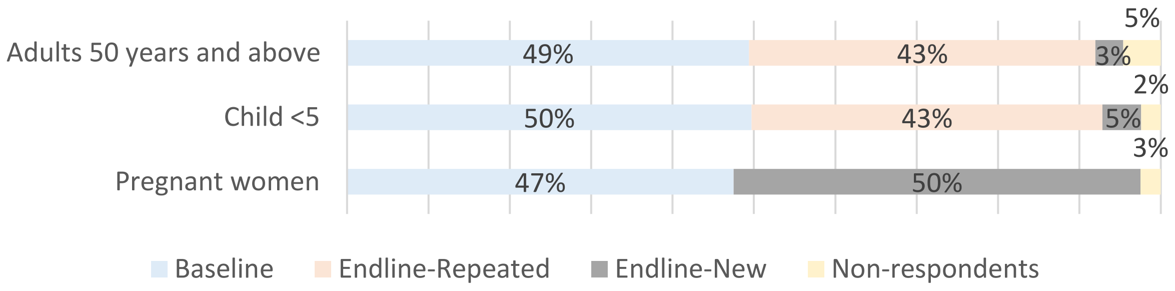

Supplemental Figure 3. Round trip time by population and site

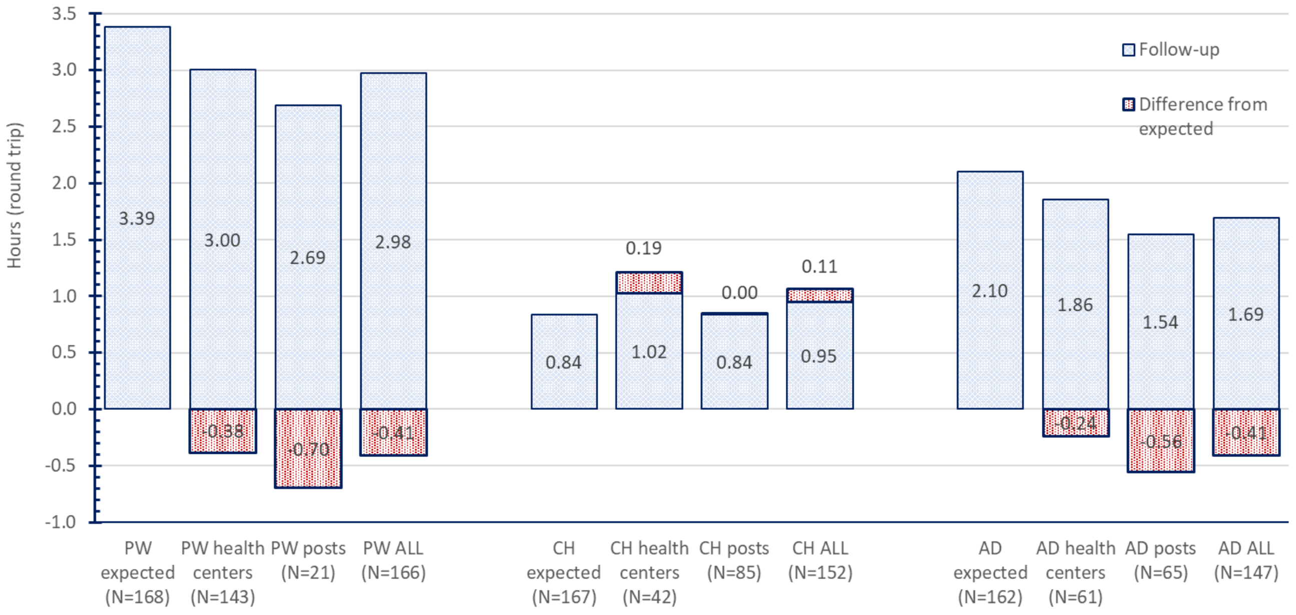

Notes: AD denotes adults 50 years and above; CH denotes child; posts denotes SGHPs; PW denotes pregnant women.

Detailed data sources

Administrative Data

We abstracted data concerning outpatient, antenatal care (ANC), and delivery utilization from health centers for patients from both the intervention and control areas before (November 2018-October 2019) and after the establishment of SGHPs (November 2019- October 2020).[1] We collected and analyzed outpatient, ANC, and delivery utilization statistics from SGHPs from November 2018 through October 2020. We adjusted the number of visits provided at the SGHPs to consider only residents of the intervention cells. This proportion from within the catchment zone was based on weekly client surveys conducted at the SGHPs between October 2019 through November 2020. This survey was conducted one day each week and collected information on several parameters, including the primary reason for visiting the post, the client's residence, and the time it took the client to reach the post. We assumed the proportion of clients from within the catchment applied equally to all diagnoses.

To estimate the impact of SGHPs on access to health care, we conducted two difference-in-difference (DID) analyses. The first analysis compared rates of outpatient visits, ANC, and deliveries within health centers to test whether SGHPs substituted for services provided by health centers. The second analyzed the overall outpatient visits per 1000 persons, ANC, and deliveries in the intervention areas (combined services from health centers and SGHPs) to examine whether SGHPs complement the health centers' efforts in the intervention areas.

Household Survey

The aim of the household surveys was to estimate change in (1) access to primary care, (2) quality of care, and (3) change in round-trip travel time in obtaining health care due to the newly established SGHPs.

We conducted baseline and end line surveys for three patient groups: mothers of children under four (at enrollment), pregnant women, and elderly adults 50 years and above. Mothers of children under four and elderly adults were re-interviewed about one year later. We identified separate cohorts of pregnant women at baseline and end line chosen from the same villages in the same way and interviewed women in each cohort only once. To collect the data on children under age five, we interviewed mothers who had children under the age of four at baseline so that children would be under age five by the end of the follow-up period, i.e., one year later. The survey questionnaires were developed in collaboration with colleagues from Rwanda and translated to Kinyarwanda. They mostly covered the utilization of health services, out-of-pocket costs, and travel details. Most questions were multiple choice, with the remainder requiring answers of a few words. The protocol, including consent forms, was submitted and subsequently approved by the ethics committees at Brandeis University and Rwanda's Ministry of Health.

A comprehensive list of households with eligible participants for each of the three patient groups (pregnant women, mothers of children under the age of four, and adults 50 years and above) was prepared by community health workers (CHWs) assigned to these populations in the selected cells and organized by the CHWs' coordinators at the district office in Nyamata. From the comprehensive list of eligible participants, investigators performed a random sampling of households/participants and selected the first seven participants from each of the three patient groups on the list. The selected members' names were given to the CHWs' coordinator at the district hospitals and health centers. CHWs invited selected participants to attend the interviews. A random sample was performed to choose participants in the nearest village or cell when a cell did not meet the needed number of participants, especially pregnant women.

Interviews took between 30 minutes to 1 hour and were administered by trained interviewers who read the questions to the participant and recorded the answer on a paper-based questionnaire. The baseline survey was conducted in person by seven trained interviewers at a central meeting point in each village in September and October 2019. The end line surveys were conducted by four interviewers over the phone due to COVID-19 restrictions in June and July 2021. The surveys were conducted in 16 cells, 8 with SGHPs and 8 in the control cells. For each patient group (pregnant women, children under 5, and elderly adults), the CHWs invited 320 individuals to be interviewed in each round for a total of 1,920 interviews.

Focus Group Discussions

We convened four focus group discussions (FGD) with CHWs operating in the eight intervention cells. Each FGD brought together CHWs from two cells, with 8 CHW participants in each group. Participants were selected based on the population they represented in the three patient groups of interest (pregnant women, children under five years, and adults 50 years and above). FGDs lasted an average of two hours, were conducted in Kinyarwanda, and were facilitated by two experienced data collectors (acting as a moderator and note-taker). Participants received 5,000 RWF (US$5.50) as travel reimbursement and compensation for the time investment. The FGD facilitator followed a guide with open-ended questions to allow CHWs to verbalize their ideas about the benefits and limitations of the SGHP introduced in their communities in 2019 for the three patient groups. FGDs were audio-recorded,

and each participant was assigned a number; the moderator referred to these numbers to call on participants during the discussion. The interviews were transcribed and translated into English. Thematic analysis was used to identify common themes for each population group and among populations.

Study Indicators

In total, we studied ten indicators. Below are the definitions of each indicator by patient type:

Pregnant women

1- Promptness in the initiation of first ANC visit reported as the number of months from last menstrual period
2- Perceived quality of care index, an index with a scale from 0 to 100 with a higher value indicating better results (average responses associated with services provided during first ANC visit: time spent with patient during the first ANC visit, provider knowledge, and provider treated patient with respect)
3- Patient-reported service index, an index with a scale from 0 to 100 with a higher value indicating better results (average number of recommended services reported during first ANC visit, including a blood test to confirm pregnancy, and lab test for malaria, HIV/AIDS, and other diseases)
4- The round-trip time for care in hours

Children Under Age 5

1- Promptness in initiating care, measured as time from illness onset until the first visit (in days)
2- Parent-reported services index, a 0-100 index with a scale from 0 to 100, with a higher value indicating better results (number of activities reported from the child's most recent visit to a health facility, e.g., provider asked the mother to describe child's need, provider examined child, and provider prescribed medication)
3- The round-trip time for care in hours

Elderly (adults 50 years and above)

1- Perceived quality index, an index with a scale from 0 to 100 with a higher value indicating better results (average responses associated with the last visit to health facility: time spent with the patient, provider knowledge, and provider treated patient with respect)
2- Patient-reported service index, an index with a scale from 0 to 100 with a higher value indicating better results (% of recommended services performed within a specified interval). These recommended services included blood pressure check and chest examination, lab blood test for diabetes, urine test, advice on diet, smoking and alcohol use, and an annual checkup.
3- The round-trip time for care in hours

Quality of care and service completeness

Quality of care

At the baseline, women in the intervention areas rated the overall quality of care (a scale from 0 to 100) at 32.0(14.6) compared to 34.2(14.0) in the control area. After the implementation of SGHPs, pregnant women in the intervention areas rated the quality of care at 30.0(1.4) compared to 24.7(11.8) in the control areas. The expected overall quality score in the intervention areas was 22.41. However, the actual quality score was 7.6 points higher compared to what would have happened without SGHPs. Increased quality of care was observed among pregnant women who utilized both the health centers and SGHPs.

For adults 50 years and above the perceived quality index, which had a baseline score of 85.9 out of 100 in the intervention areas, deteriorated (by -1.97 points) in intervention areas compared to what would have happened without SGHPs. The deterioration in health centers was observed to be -6.51 points, while the quality of services received at SGHP increased by 2.12 points (Figure 4).

Supplemental Figure 4. Change in the quality index by population and site

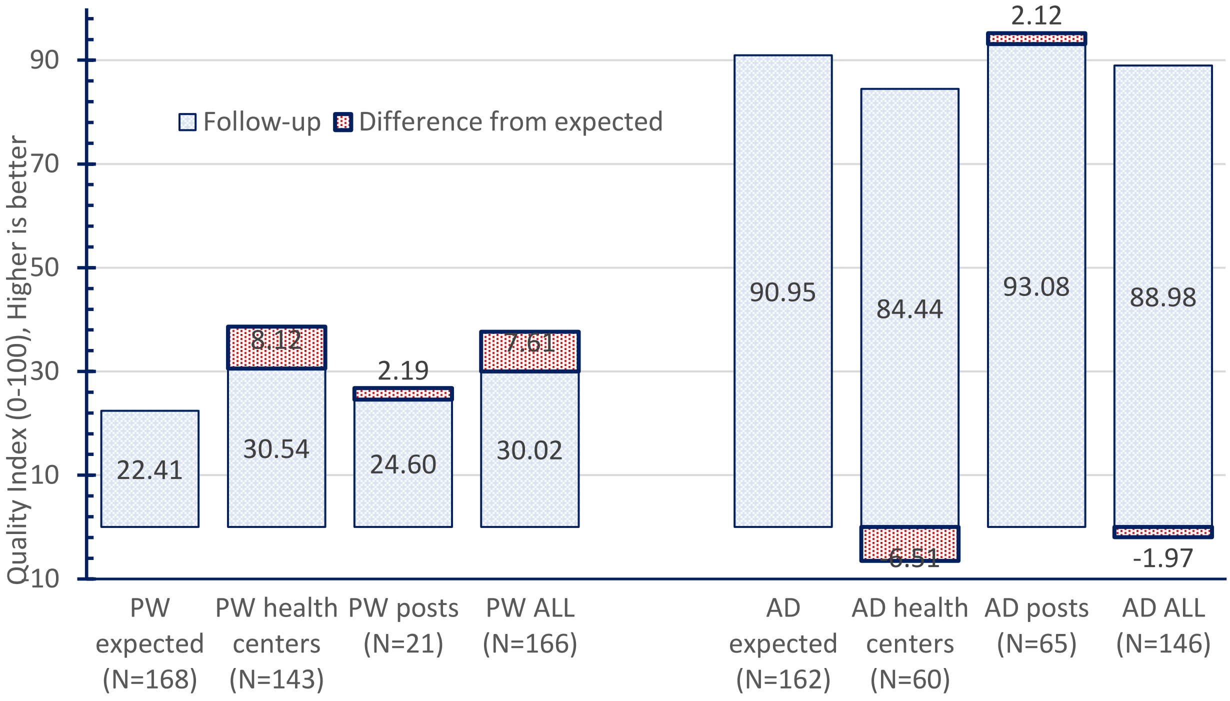

Notes: AD denotes adults 50 years and above; posts denotes SGHPs; PW denotes pregnant women.;

Health care service completeness and intensity

At the baseline, the average service intensity score (services per visit) for pregnant women in the intervention areas was 60.2(17.3) compared to 69.8(15.6) in the control area. After implementing SGHPs, the service intensity score for pregnant women in the intervention and control areas deteriorated to 51.5(15.1) and 58.8(13.9), respectively. The expected service index score in the intervention areas for pregnant women was 49.3 (Figure 5). However, the actual service score was 2.3 points higher compared to what would have happened without SGHPs. The increases in the service index were higher in the SGHPs (9.1 points) compared to the health centers (1.5).

Supplemental Figure 5. Change in the service index by population and site

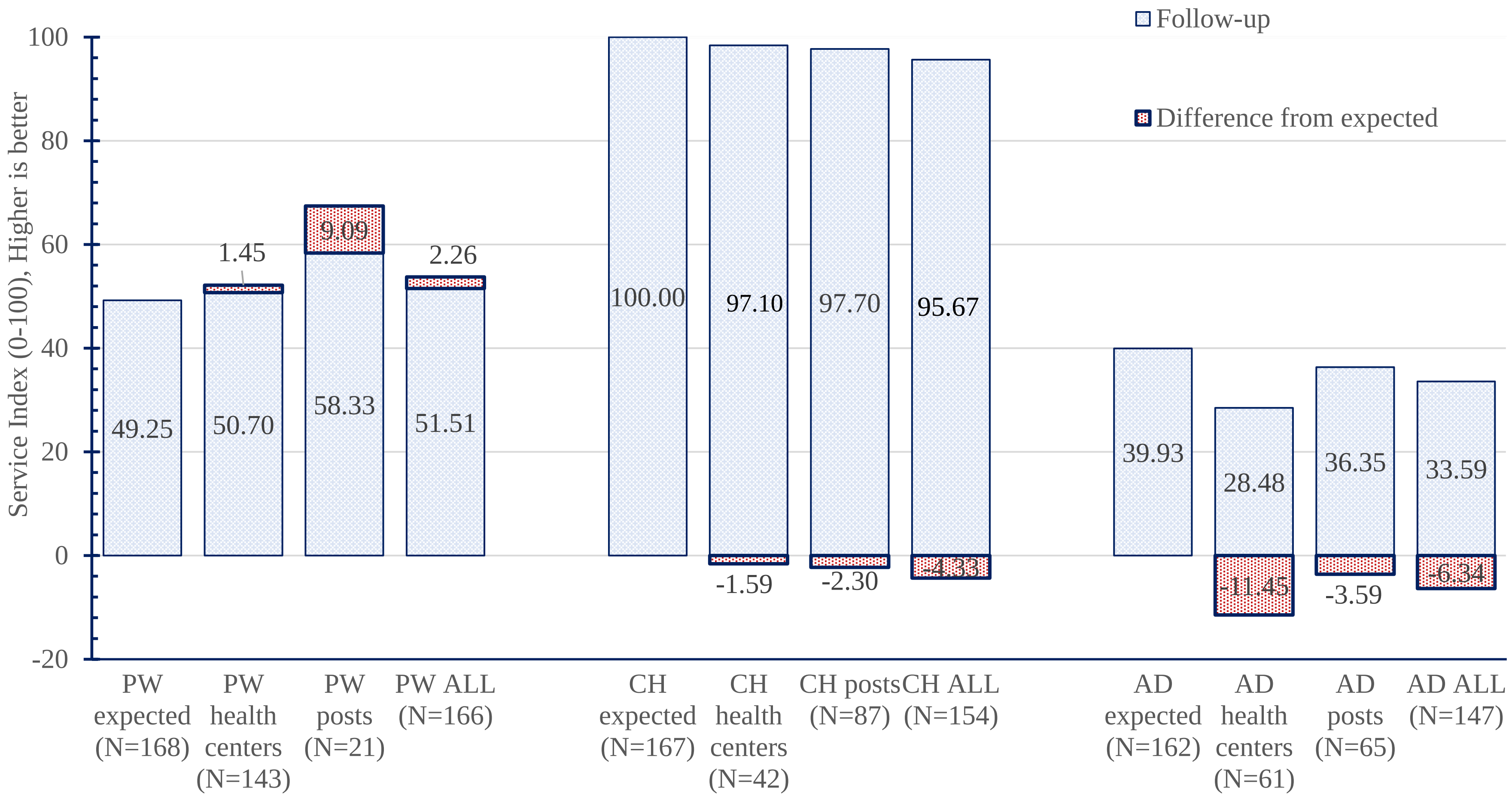

Notes: AD denotes adults 50 years and above; CH denotes child; posts denotes SGHPs; PW denotes pregnant women.;.

At the baseline, the average service score for children under age 5 in the intervention areas was very high at 97.9(12.3) compared to 95.0(11.9) in the control area. After implementing SGHPs, the service score for children in the intervention deteriorated to 95.7(13.0) but improved in the control areas 97.4(9.0). The expected service index score for children in the intervention areas was 100 (Figure 5). However, the actual service score decreased by 4.3 points compared to what would have happened without SGHPs. The reduction was seen in both the SGHPs (2.3 points) and the health centers (1.6).

At the baseline, the average service score for the elderly adults 50 years and above in the intervention areas was 30.0(22.3) compared to 34.7(25.8) in the control area. After the implementation of SGHPs, the service score for elderly adults improved in both the intervention 33.6(28.2) and control areas 44.7(30.2). The expected service index score in the intervention areas for adults was 39.9 (Figure 5) However, the actual service score decreased by 6.34 points compared to what would have happened without SGHPs. The reduction in the service score was seen in both the SGHPs (3.6 points) and the health centers (11.5).

Interpretation from Focus Group Discussions

Pregnant women

Community health workers were asked about ANC services provided at the SGHPs. Several themes that emerged from the FGD include pregnant women's preference to attend ANC at health centers due to the center's capacity to provide the needed care; pregnant women's desire for continuity of care; lack of care coordination between the health centers and SGHPs, highlighted by health centers' refusal to treat women seen at SGHPs; and the incentives to CHW and pregnant women to attend health centers. For CHWs, they receive incentives to refer pregnant women to a health center but not to SGHPs. For pregnant women, attending a health center is associated with benefits, such as getting free insecticide treated nets, and in some cases, they might receive subsidies for food and cash. These benefits are not available at the SGHPs. However, some women found refuge in the SGHPs from paying a fine to the health center for delay in attending the first ANC visit. This might explain the difference in the average delay in attending the first ANC visits in the health centers compared to the SGHPs.

Pregnant women's perception of care at both health centers and at SGHPs was low. This is due for several reasons including, the low number of staff at SGHPS, and the high provider turnover in both health centers and SGHPs, affecting pregnant women's sense of familiarity with health providers and the continuity of care. Moreover, pregnant women prefer to see experienced providers rather than nursing students doing their clerkship at the health center, who they perceive as less knowledgeable.

The restrictions associated with the COVID-19 pandemic and the shortage and movement of medical staff had a negative impact on pregnant women's perception of the quality of care provided at both health centers and SGHPs due to lack of familiarity, and occasional unwillingness of new providers to work with the CHW to meet the pregnant woman's needs.

Children under age five

When a child gets sick, the parents might seek the advice of a CHW, traditional healer, or go to the pharmacy for medication before they take the child to the health center. This would generally apply for those who do not have health insurance. For this population, seeking care through a CHW is affordable and could be free if the family falls within the *Ubudehe* categories 1 and 2 (lower socio-economic strata). In addition, financial constrains were reported at the SGHPs where providers urge

parents to buy medication not covered by the health insurance at a higher price, discouraging parents from seeking SGHPs for future care and reducing the promptness of seeking care from the illness onset.

Adults 50 years and above

Quality of care was associated with the capacity within the health care facilities. According to the CHWs, some adults didn't perceive the quality of care at the SGHPs as good as the health centers because of the number of staff available at the SGHPs, and the fact that some of their needs, such as need for dental care or management of chronic conditions, were not met at the SGHP level. Again, the affordability of care was raised. The CHWs noted that SGHPs act like a private facility, asking patients to pay for much of the services including drugs, even if the drug is covered through their insurance plan and can be obtained for free at the health center. The additional cost might demolish the favorability of SGHPs closer location to patients.

COVID-19

With the advent of COVID-19 came decreased access to health care in these communities. Personal protection equipment, such as mask and gloves, were available for the CHWs. However, fear of infection reduced CHWs availability and patient utilization of their services. CHWs expected patients to seek care at the SGHPs or health centers, but the decline in health providers' availability extended to both the SGHPs and health centers. Both facilities reduced the number of staff resulting in increased waiting time for patients, required patients to use mobile money instead of cash (limiting access to those who do not have mobile money or don't know how to use it), and requested that pregnant women be tested for COVID-19 before attending their ANC visits, thus discouraging pregnant women from attending these services.

Comparative options for strengthening access to primary care

In two FGDs, CHWs mentioned some women, 3-4 women per month, delivered at SGHPs. This occurred due to the rapid labor onset which would not allow the women to seek care at the health center due to time to travel and fearing they would end up giving birth on route. For women living in remote areas, several options deserve study for prompt access to safe delivery sites. Those options include: (1) constructing SGHPs with maternity wards, similar to the four serving the more remote parts of Bugesera District, (2) building a maternity home adjacent to the health center where women can come with a companion, rest, and receive food until they go into labor, (3) arranging for ambulance transfer to pick up the pregnant woman at her home and bring her to the health center, billing the *mutuelle* at the standard rate of 400 RWF per kilometer, or (4) arranging for a network of motorcycles already in the cell that can provide emergency transport at a pre-arranged rate.

The SGHPs have shown their value for outpatient care. Between November 2019 and October 2020, the majority of services utilized at the SGHPs focus on acute conditions, with 76% focusing on care for acute respiratory diseases, intestinal parasites, and simple malaria, similar to health centers. However, SGHPs recorded no visits for chronic diseases such as HIV, hypertension or anemia, and few visits primarily for preventive care, such as ANC visits and immunization. CHWs offer limited services focusing on care for malaria, whereas SGHPs have greater capabilities, such as offering immunizations or ANC visits.

Patients of who receive care at an SGHPs rather than from a CHWs benefit from being treated in a facility with greater capability to address most acute illnesses, can receive preventive services and case

management for chronic diseases such as hypertension and diabetes, and can be treated during longer hours. Patients were prepared to make required co-payments for curative care and *mutuelles* paid most of the operating costs, so SGHPs seem financially viable.

However, the delay in payment from the community-based health insurance *mutuelle* led to some unfavorable practices to sustain SGHPs operations and pay staff, such as pushing patients to buy more medications, some of which are covered by *mutuelle* and offered for free at the health centers. This challenge could be addressed by: (1) allowing SGHPs to contract with other insurers to expand the financial pool for the SGHPs, (2) ensuring the *mutuelle* is paying SGHPs its due promptly, and (3) arranging with the government to cover the full cost of care.

Currently, SGHPs are not reimbursed for the full cost of the services provided at their facilities. For example, to treat a malaria case, a provider at the SGHP would consult, request a lab test and prescribe drugs to patients, but is reimbursed only for the consultation and drugs. To ensure good professional standards at the SGHPs, the District Health Office might consider expanding the pay-for-performance arrangement to the SGHPs and audit the prescription records on a sample basis to see whether the treatment is consistent with the diagnosis as a measure of quality of care.

SGHP's contributions to improving access to healthcare can usefully be complemented by refinements Rwanda's program of CHWs. The use of decision support systems through smart phones and arranging chats with staff at, say, the district office, may help CHWs with difficult cases. Improved and subsidized transportation is another option. Some countries use voucher systems for deliveries that include the cost of a taxi ride to a health facility for a delivery.[2] Such ideas could be extended for other life-threatening conditions, such as severe malaria or respiratory infections that threaten breathing.

In conclusion, this evaluation has shown that SGHPs have increased access to primary care for treating routine health problems and have operated as financially viable public-private partnerships. Several improvements could allow SGHPs to make greater contributions to Rwanda's health goals: strengthening linkages and referrals with CHWs and health centers, encouraging women to use SGHPs for antenatal care after their first visit, extending performance-based financing for SGHPs to better manage chronic conditions and improve quality of care, and arranging chats with medical personnel at health centers or district hospitals for advice about difficult cases.

References

1. Musange SF. Health Service Utilization In Second Generation Health Posts Catchment Areas and Matched Cells (Final Report): Prepared for Abbott, Inc. Kigali: National University of Rwanda; 2021.
2. Alfonso YN, Bishai D, Bua J, Mutebi A, Mayora C, Ekirapa-Kiracho E, 2015. Cost-effectiveness analysis of a voucher scheme combined with obstetrical quality improvements: quasi experimental results from Uganda. Health Policy Plan 30(1):88-99.
